# Rising antimicrobial resistance in urinary tract infections in Türkiye: an urgent need for update on regional treatment strategies and for enhanced antimicrobial stewardship efforts

**DOI:** 10.1007/s10096-026-05506-4

**Published:** 2026-04-15

**Authors:** Anı Akpınar, Cansel Vatansever, Şöhret Aydemir, Duygu Öcal, Elif Aktaş, Barış Otlu, Gizem Karahan, İlknur Kaleli, Jale Boral, Hayriye Altunay, Emel Uzunoğlu, Sebahat Aksaray, İlkay Nur Can, Selin Kolsuz, Özlem Kurt Azap, Önder Ergönül, Füsun Can

**Affiliations:** 1 Koç University İşbank Center for Infectious Diseases (KUISCID), İstanbul, 34010 Türkiye; 2 Faculty of Medicine, Department of Medical Microbiology, Ege University, İzmir, Türkiye; 3 School of Medicine, Department of Medical Microbiology, Ankara University, Ankara, Türkiye; 4 Hamidiye Etfal Training and Research Hospital, Department of Medical Microbiology, University of Health Sciences, İstanbul, Türkiye; 5 Faculty of Medicine, Department of Medical Microbiology, İnönü University, Malatya, Türkiye; 6 Department of Infectious Diseases, Koç University Hospital, İstanbul, Türkiye; 7 Faculty of Medicine, Department of Medical Microbiology, Pamukkale University, Denizli, Türkiye; 8 Department of Infectious Diseases, Başkent University, Adana, Türkiye; 9 Faculty of Medicine, Department of Medical Microbiology, Giresun University, Giresun, Türkiye; 10 Haydarpasa Numune Training and Research Hospital, Department of Medical Microbiology, University of Health Sciences, İstanbul, Türkiye; 11 Şanlıurfa Numune Training and Research Hospital, Şanlıurfa, Türkiye; 12 School of Medicine, Koç University, İstanbul, Türkiye; 13 School of Medicine, Department of Infectious Diseases, Başkent University, Ankara, Türkiye; 14 School of Medicine, Department of Infectious Diseases, Koç University, İstanbul, Türkiye; 15 School of Medicine, Department of Medical Microbiology, Koç University, İstanbul, Türkiye; 16 Present address: Department of Translational Medicine, Clinical Microbiology, Lund University, Malmö, Sweden

**Keywords:** Antibiotic resistance, UTI, antimicrobial stewardship, *Klebsiella pneumoniae*, *Escherichia coli*, Antibiotic use

## Abstract

**Purpose:**

Türkiye ranks among the highest in antibiotic consumption within OECD countries and faces growing challenges from multidrug-resistant uropathogens. Nationwide, resistance-guided treatment data remain scarce. The main objective of this work is to provide a comprehensive nationwide data on antibiotic resistance patterns among uropathogens to guide treatment options in urinary tract infections (UTIs).

**Methods:**

A multicenter, retrospective observational study was conducted between 2021 and 2023 across 11 centers from 8 provinces representing all major geographical regions of Türkiye. Uropathogens isolated from adult patients with UTIs were analyzed to assess temporal trends and regional variability in antimicrobial resistance.

**Results:**

In outpatients, resistance to commonly used oral agents (amoxicillin–clavulanate, ciprofloxacin) frequently exceeded 30%, peaking above 60% in Eastern and Southeastern regions. Nitrofurantoin and fosfomycin consistently demonstrated low resistance nationwide, supporting their continued use for uncomplicated UTIs. Aminoglycosides, including amikacin, maintained low resistance rates across all regions, indicating potential for empirical use, though gradual year-on-year increases were observed. Multidrug and extensively drug-resistant isolates were most prevalent among elderly and inpatient populations. In inpatients, *Klebsiella pneumoniae* showed particularly high resistance, with the last-line agent ceftazidime–avibactam resistance exceeding 60% in some regions.

**Conclusion:**

For the treatment of uncomplicated UTIs, nitrofurantoin and fosfomycin remain reliable options, while aminoglycosides may be considered for complicated UTIs with caution. Pregnant women and elderly patients require special consideration due to limited alternatives. In inpatient settings, aminoglycosides remain viable empirical choices for suspected MDR infections. Strengthening antimicrobial stewardship and surveillance is critical to improving UTI management and combating antibiotic resistance in Türkiye.

## Introduction

Urinary tract infections (UTIs) are a common cause of outpatient visits and hospitalizations. Recent epidemiological analyses indicate that the global annual incidence of UTIs reached 404.6 million cases in 2019, with a continued upward trend through 2021 [1, 2]. Besides, antimicrobial resistance reached an alarming level in UTI pathogens as a consequence of inappropriate and widespread use of antibiotics. The success of empirical therapy has become challenging due to high antibiotic resistance rates.

In 2019, UTIs ranked 4th among deaths due to antibiotic resistance in the world and 64,890 deaths were directly related to resistant UTI infections [3, 4]. Moreover, multidrug or pandrug resistant uropathogens are emerging all over the world. Recent studies have documented the emergence of multidrug-resistant (MDR) bacterial pathogens in UTIs from diverse geographic and clinical sources [5]. The most common MDR uropathogens include *Escherichia coli*,* Klebsiella pneumoniae*, and other *Enterobacterales*, with resistance patterns varying by region, age, and sex. For example, resistance rates to commonly used antibiotics such as ampicillin/amoxicillin, trimethoprim, and fluoroquinolones now frequently exceed 50% in many populations [6, 7] and MDR rates among uropathogens can reach 65–71% in low-resource settings [6, 8, 9]. These alarming rates underscore the critical importance of antimicrobial stewardship efforts—both to reduce resistance rates and restore the effectiveness of existing treatments in high-use settings such as Türkiye, and also to preserve low resistance levels and sustained treatment efficacy in settings with more favorable resistance profiles.

An improved understanding of current nationwide resistance patterns can help inform empirical treatment and antimicrobial stewardship efforts. Türkiye has the second highest antibiotic consumption among OECD countries [10]. But unfortunately, there is no comprehensive study evaluating the UTI pathogens and antimicrobial resistance in Türkiye in recent years. Strict regulations have been implemented by the Ministry of Health that restrict the use of antibiotics without prescription. Nevertheless, in current practice, UTIs are often managed empirically in primary care settings, without urine cultures. In hospital settings, although urine cultures are more routinely performed, the widespread use of broad-spectrum antibiotics has contributed to high antimicrobial resistance rates, thereby limiting available treatment options. The lack of data leads to a critical uncertainty about the strategies and practices to be developed for the treatment of UTIs and prevention of rising AMR in uropathogens in Türkiye.

We aimed to determine the most common UTI pathogens and antimicrobial resistance rates in outpatients and inpatients to identify nationally and regionally preferable antibiotics for the empirical treatment of UTIs.

## Methods

### Data collection and processing

A total of 11 centers from 8 provinces representing Türkiye’s seven geographical regions participated in the study. These centers belonged to 8 subregions of 7 NUTS-1 regions described in Nomenclature of Territorial Units for Statistics (NUTS): two centers from İstanbul Region (İstanbul, TR100), three centers from West Anatolia Region (Ankara, TR510), two centers from Aegean Region (İzmir, TR310; Denizli, TR322), one center from East Black Sea Region (Giresun, TR903), one center from Mediterranean Region (Adana, TR621), one center from Central East Anatolia Region (Malatya, TRB11) and one center from Southeast Anatolia Region (Şanlıurfa, TRC21). Demographic and microbiological data were extracted from the hospital information systems of participating centers between January 2021 and January 2024. The dataset comprised urine culture results from patients aged ≥ 18 years who presented to outpatient clinics or were hospitalized with a recorded preliminary diagnosis of urinary tract infection. Repeat urine samples obtained within 15 days were excluded from the analysis.

Pathogen identification and susceptibility testing were performed by conventional methods and automated systems. Antibiotic resistance rates were calculated for the most common Gram-negative uropathogens, including *E. coli*, *K. pneumoniae* and *P. aeruginosa*. Intermediate susceptibility results were interpreted as “susceptible, increased exposure (I)” according to EUCAST and treated as susceptible in analyses where appropriate exposure could be achieved. The results were interpreted according to the European Committee [11]. Multidrug resistance (MDR) and extensive drug resistance (XDR) were defined according to internationally accepted criteria [12]. MDR was defined as resistance to ≥ 3 of the following antimicrobial categories: (1) Colistin, (2) Nitrofurantoin, (3) Trimethoprim-Sulfamethoxazole, (4) fluoroquinolones (Ciprofloxacin), (5) Amoxicillin-Clavulanate, (6) Piperacillin/tazobactam, (7) carbapenems (Ertapenem or Meropenem), (8) third-generation cephalosporins or β-lactam/β-lactamase inhibitor combinations (Ceftriaxone or Ceftazidime–avibactam), and (9) aminoglycosides (Amikacin or Gentamicin). XDR was defined as resistance to ≥ 6 of these antimicrobial categories. Since the standards were only defined for *E.coli*, we only presented the resistance results of *E. coli* for Fosfomycin [11]. For colistin and ceftazidime-avibactam, resistance rates were calculated only among carbapenem-resistant isolates.

### Statistical analysis

A multivariate logistic regression model was implemented in Python using the statmodels library to assess the changes in resistance rates over the years. The dependent variable was resistance status (resistant vs. susceptible). Year was included as an independent variable to evaluate changes over time. The model was adjusted for age, gender, and geographic region. Odds ratios (ORs) and 95% confidence intervals (CIs) were calculated, and results were visualized using GraphPad Prism.

## Results

Among the Gram-negatives, *E. coli* was the most frequently isolated pathogen from both outpatients (56%) and inpatients (37%, Table 1), followed by *K. pneumoniae* (12% and 17%) and *P. aeruginosa* (3% and 5%). Other Gram-negative genera comprised 8% and 11% of pathogens isolated from outpatients and inpatients. *Enterococcus* spp. was the most common among Gram-positive pathogens, with isolation rates of 10% in outpatients and 17% in inpatients. The remaining Gram-positive uropathogens were *Streptococcus* spp (4% and 1%), *Staphylococcus* spp (4% and 3%) and ≤ 1% other genera in outpatients and inpatients.

**Table 1:** Comparison of antibiotic resistance rates between inpatient and outpatient bacterial isolates (%). Total column indicates all uropathogens

Bacteria Antibiotic	E. coli (*n* = 50,170)		K. pneumoniae (*n* = 15,077)		*P*. aeruginosa (*n* = 4,340)		Total (*n* = 105,934)	
	Outpatient (*n* = 17,267)	Inpatient (*n* = 32,903)	Outpatient (*n* = 8,043)	Inpatient (*n* = 7,034)	Outpatient (*n* = 2,597)	Inpatient (*n* = 1,743)	Outpatient (*n* = 47,237)	Inpatient (*n* = 58,697)
AMP	64.71	76.95	NA	NA	NA	NA	61.03	NA
AMC	35.98	NA	46.01	NA	NA	NA	39.44	NA
PPT	NA	17.72	NA	54.89	NA	30.9	NA	30.25
CTR	33.12	48.16	46.05	66.99	NA	NA	34.46	52.22
CTZ	35.21	NA	46.11	67.73	30.68	38.73	35.83	51.28
CZA*	NA	NA	NA	40.11	NA	48.72	NA	41.54
MEM	NA	1.16	NA	33.32	NA	23.78	NA	14.9
IMI	NA	1.89	NA	29.35	NA	29.86	NA	16.6
ETP	NA	5.04	NA	41.29	NA	NA	NA	16.09
CIP	37.61	49.62	42.11	60.75	24.7	27.86	36.85	51.01
AMI	3.15	3.55	9.77	25.74	6.6	12.02	4.73	12.84
GEN	13.83	19.83	17.27	33.32	9.4	45.54	15.08	29.02
TMP-SMX	34.86	44.29	38.01	53.01	36.71	NA	32.72	44.33
COL*	NA	NA	NA	34.68	NA	7.36	NA	21.6
FOS	2.65	3.5	NA	NA	NA	NA	NA	NA
FUR**	2.68	2.84	41.41	NA	NA	NA	5.74	NA
MDR	29.06	42.16	41.43	63.17	9.25	18.35	25.91**	37.18
XDR	2.05	5.12	14.54	36.81	0.06	0.08	3.47	10.12

*AMC* Amoxicillin-Clavulanate, *AMI* Amikacin, *AMP* Ampicillin, *CIP* Ciprofloxacin, *COL* Colistin, *CTR* Ceftriaxone, *CTZ* Ceftazidime, *CZA* Ceftazidime-avibactam, *ETP* Ertapenem, *FOS* Fosfomycin, *FUR* Nitrofurantoin, *GEN* Gentamicin, *IMI* Imipenem, *MEM* Meropenem, *PPT* Piperacillin/tazobactam, *TMP-SMX* Trimethoprim-Sulfamethoxazole, *NA* No data available

* The rate is for carbapenem resistant isolates

**Total rate is for *E. coli* and *K. pneumoniae*

**Fig. 1:**
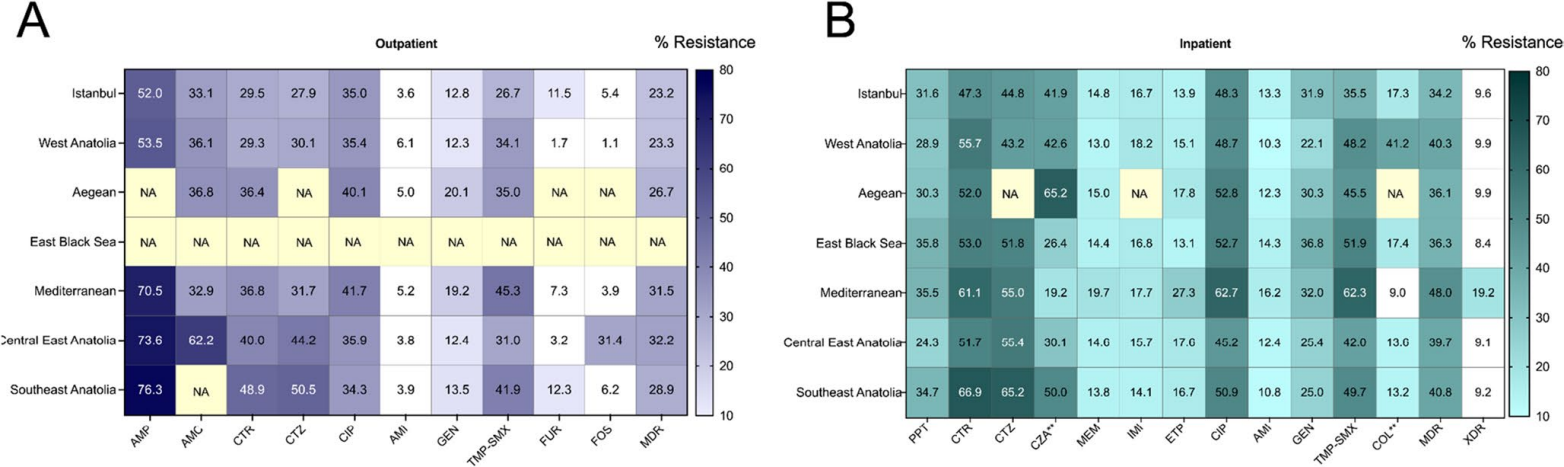
Regional distribution of antibiotic resistance rates (%) among all uropathogens isolated from outpatients (**A**) and inpatients (**B**). Rows indicate NUTS-1 regions. Pale yellow areas indicate data not available for the corresponding region–antibiotic combination (NA: No data available). Color bars represent resistance rates, ranging from light (lowest) to dark (highest). **Colistin and ceftazidime-avibactam resistance rates were calculated only among carbapenem-resistant isolates

In general, resistance rates for first-line oral antibiotics were over 30%, exceeding the critical threshold of 20%, above which expert consensus is to reconsider the empirical use of an antibiotic, due to increased risk of inappropriate initial therapy and treatment failure. The rates varied across different regions (Fig. 1). Among outpatient isolates, ciprofloxacin and trimethoprim-sulfamethoxazole resistance were over 30% across the country. Resistance to penicillin-class antibiotics exceeded 60% in the Mediterranean, Central East Anatolia, and Southeast Anatolia regions. Furthermore, resistance to third-generation cephalosporins, specifically ceftriaxone (48.8%) and ceftazidime (50.4%), were also highest in the Southeast Anatolia region (Fig. 1A). Among inpatient isolates, the rates were significantly higher compared to those isolated from outpatients. In all provinces, resistance to amikacin and carbapenems remained below the 20% threshold overall, except for *P. aeruginosa* and *K. pneumoniae*, where carbapenem resistance exceeded this threshold. However, resistance rates to other antibiotics—such as ciprofloxacin (51%), ceftazidime (51%), ceftriaxone (52%), and trimethoprim-sulfamethoxazole (44%)—exceeded 60% in Southeast Anatolia and Mediterranean regions (Fig. 1B).

Among all uropathogens, *K. pneumoniae* exhibited the highest resistance against most antibiotic classes. In outpatient *K. pneumoniae* isolates, nitrofurantoin and ciprofloxacin resistance rates were over 40% and 41.4% of isolates exhibited MDR profile (Table 1). In inpatients, the resistance rates were at the alarming level particularly for ceftriaxone (66.9%), ceftazidime (67.7%), ciprofloxacin (60.7%) and, to a lesser extent, carbapenems (29–41%). The ceftazidime-avibactam resistance was 40% and reached over 60% in the Aegean region. Amikacin and gentamicin resistance were below 20% in outpatient isolates but 25.7% and 33.3% of the inpatient isolates were resistant to these antibiotics. MDR and XDR rates were also highest in *K. pneumoniae* from inpatients (63.1% and 36.8%, respectively).

*E. coli* isolates showed low and stable resistance below 4% against fosfomycin, nitrofurantoin and amikacin from both outpatient and inpatients. However, the resistance for beta-lactams and ciprofloxacin were above 30% (Table 1).

*P. aeruginosa* isolates from outpatients had only resistance below 20% against amikacin and gentamicin. The ciprofloxacin resistance was 24.7% in outpatient isolates. In inpatient isolates, resistance rates below 20% were observed for amikacin and colistin. There was a significant difference in gentamicin resistance between the isolates from outpatients and inpatients (9.4% vs. 45.5%) (Table 1).

**Fig. 2:**
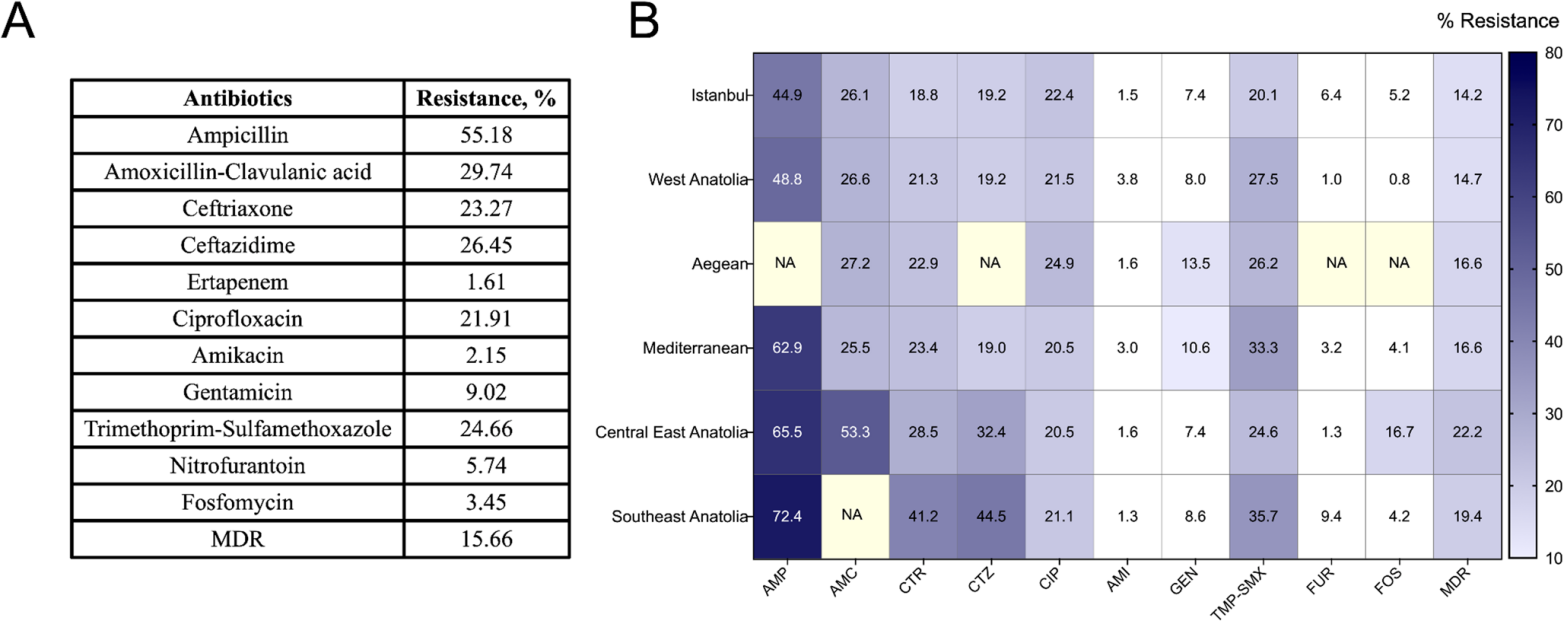
**A** Antibiotic resistance rates (%) among all uropathogens isolated from female outpatients aged 18–45 years **B** Regional distribution of antibiotic resistance rates (%) among female outpatients aged 18–45 years for all uropathogens. Pale yellow areas indicate data not available for the corresponding region–antibiotic combination (NA: No data available)

Among women aged 18–45 years, the nitrofurantoin resistance was 5.7%, but resistance to amoxicillin-clavulanate was observed to exceed the internationally accepted 20% threshold across all provinces and was 53.2% in the Central East Anatolia region (Fig. 2). In parallel to the general population, resistance to ceftriaxone (41.1%) and ceftazidime (44.4%) were high in the Southeast Anatolia region (Fig. 2).

**Fig. 3:**
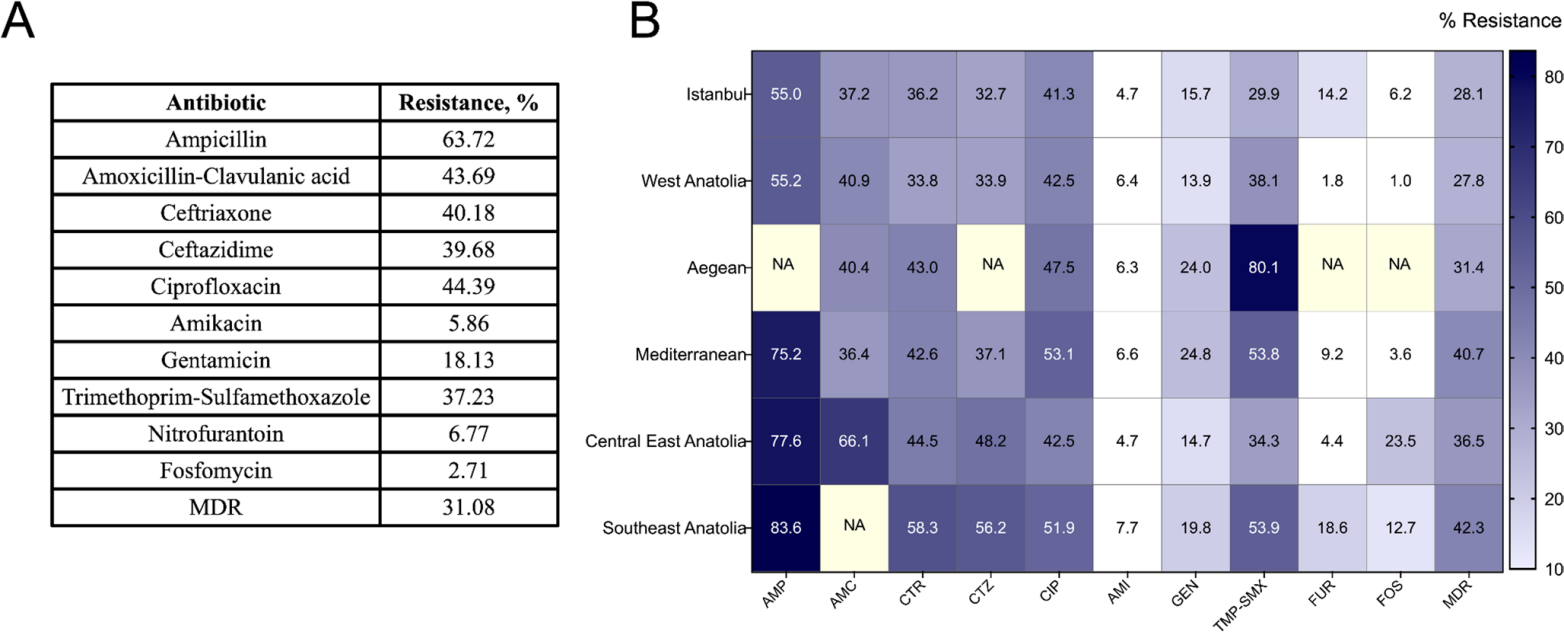
**A** Antibiotic resistance rates (%) among outpatients aged over 65 years **B** Regional distribution of antibiotic resistance rates (%) among outpatients aged over 65 years. Data is given for all uropathogens. Pale yellow areas indicate data not available for the corresponding region–antibiotic combination (NA: No data available)

Among outpatients aged over 65 years, the resistance rates against all antibiotics were higher than the general population (Fig. 3). The ciprofloxacin resistance was 44.4%, amoxicillin-clavulanate was 43.7%, trimethoprim-sulfamethoxazole resistance was 37.2%. In this age group, resistance to nitrofurantoin and amikacin was the lowest, remaining below 20%. The MDR profile was also higher than the general population, exceeding 40% in the Southeast Anatolia and Mediterranean regions (42.3% and 40.7%, respectively; Fig. 3).

**Fig. 4:**
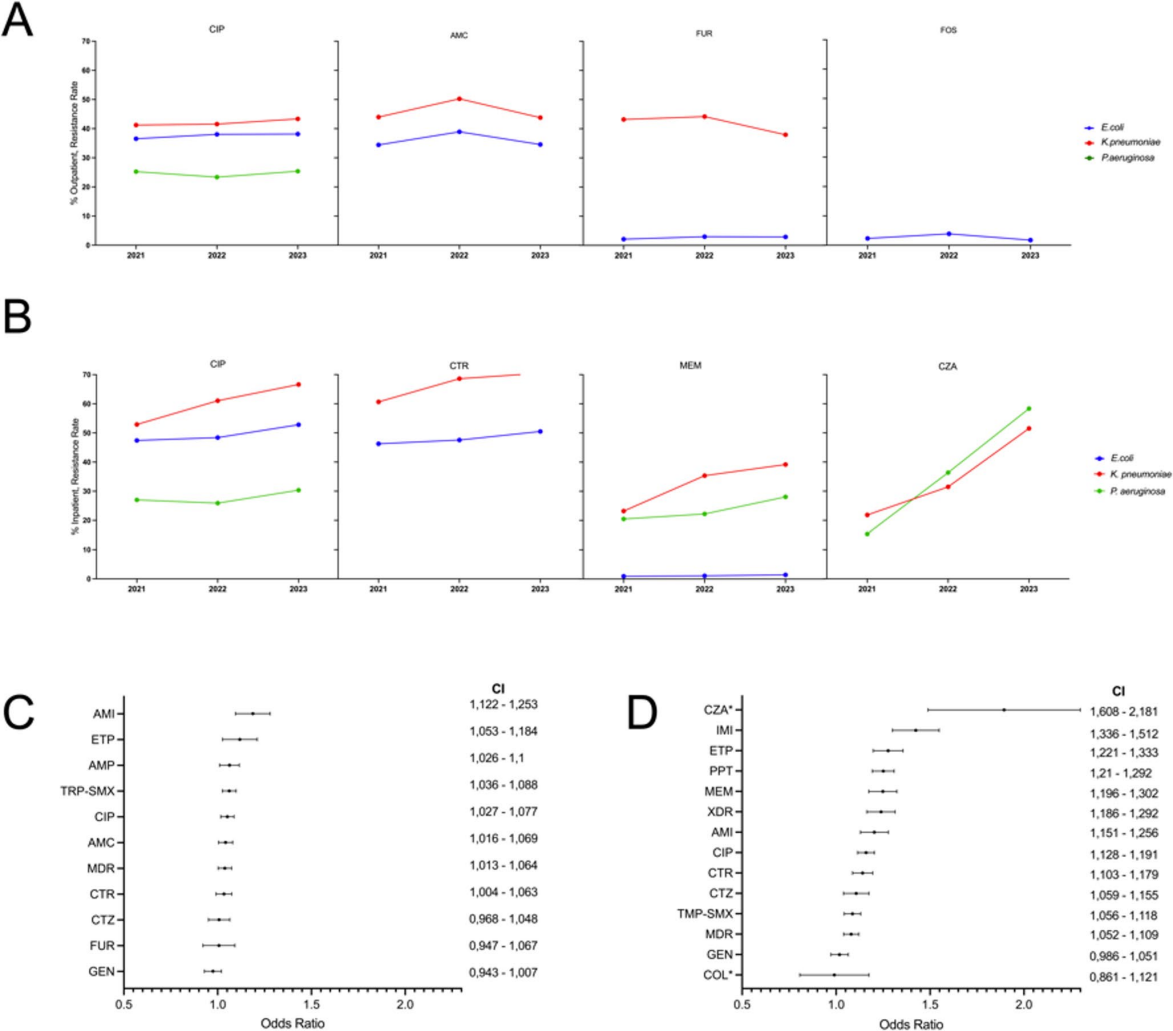
Year-on-year resistance rates (%) of selected antibiotics in uropathogens *E. coli*, *K. pneumoniae*, and *P. aeruginosa* in **A** outpatients and **B** inpatients. Antibiotic resistance increase risk from 2021 to 2023 in **C** outpatient and **D** inpatient groups

Analysis of year-on-year changes in antibiotic resistance demonstrated an overall increase in resistance rates for all three pathogens, particularly in *K. pneumoniae* (Fig. 4A and B). In a multivariate logistic regression analysis adjusted for age, gender, and region, year was associated with increasing odds of resistance. Among outpatient isolates, the risk of increase in resistance was especially significant for amikacin and ertapenem (OR: 1.186 and 1.117; Fig. 4C). Among inpatient isolates, the high risk of increase in ceftazidime-avibactam resistance is particularly concerning (OR: 1.872; Fig. 4D). Furthermore, carbapenem resistance rates, already notably high in this patient population, continued to exhibit an upward temporal trend.

**Fig. 5:**
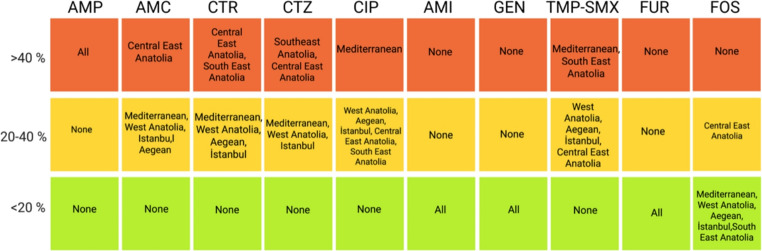
Recommended antibiotic use for outpatients based on regional resistance levels. Red (> 40%) Not Recommended — High resistance in the listed region(s); avoid use. Yellow (20–40%) Use with Caution — Moderate resistance; consider alternatives or perform susceptibility testing. Green (< 20%) Recommended — Low resistance; suitable for empirical treatment. All: all geographic regions, None: No regions

An evaluation of empirical outpatient treatment options was performed using regional antimicrobial resistance data. Resistance rates were stratified into three categories: high (> 40%), moderate (20–40%), and low (< 20%), and treatment recommendations were developed accordingly (Fig. 5). Based on this framework, nitrofurantoin and fosfomycin were the most suitable first-line agents nationwide due to consistently low resistance levels. Amikacin and gentamicin were identified as highly effective parenteral agents, appropriate when oral therapy is not feasible. In contrast, ampicillin demonstrated high resistance rates in all regions and should be avoided.

## Discussion

UTIs remain a significant public health concern. A comprehensive knowledge on the epidemiology of uropathogens, along with antibiotic resistance patterns are critical to predict treatment outcomes and guide clinical decision making. In this retrospective observational study, we presented the burden of antimicrobial resistance among uropathogens isolated from both outpatients and inpatients in Türkiye, a country with high antibiotic consumption rates, and assessed the effectiveness of empirical therapy options recommended by guidelines.

The Turkish Ministry of Health’s guideline, Rational Use of Antibiotics in Adult Patients, recommends trimethoprim-sulfamethoxazole, nitrofurantoin, and fosfomycin for treating cystitis but does not indicate the first line preference [13]. The high resistance to trimethoprim-sulfamethoxazole, limits its use in Türkiye. In a recent consensus report and in international guidelines, nitrofurantoin is recommended as the first-line oral treatment for simple cystitis [14]. In parallel to this statement, the low resistance to nitrofurantoin across all regions in Türkiye suggested that it is also an appropriate treatment option for outpatients with low risk of MDR infections, such as those without a history of frequent antibiotic use or prior hospitalizations. However, nitrofurantoin is commonly used for the prevention of recurrent urinary tract infections (RUTIs), including long term continuous or intermittent antibiotic prophylaxis [15, 16]. In Türkiye, long-term prophylactic use of nitrofurantoin, especially for RUTIs is common and has been reported in earlier studies [17]. A recent meta-analysis reported no significant difference in prophylactic efficacy between intermittent and continuous use of nitrofurantoin [15]. The overuse of nitrofurantoin must be strictly controlled to preserve its efficacy as an empirical therapy. Clinicians should also assess renal function and relevant comorbidities before prescribing nitrofurantoin and consider the limited activity of nitrofurantoin against non-*E. coli* isolates.

With low resistance rates across all regions, fosfomycin appears to be a reliable option for empirical treatment of outpatients in Türkiye. Some national guidelines, like France guideline for treatment of UTI, recommend fosfomycin as a first-line empirical antibiotic [18]. However, a randomized clinical trial conducted in 2018 reported that its 14-day clinical response was lower compared to nitrofurantoin [19], indicating that favorable resistance profiles may not necessarily translate into equivalent clinical efficacy. Furthermore, the recently published consensus report recommends the use of fosfomycin if there is a risk of MDR pathogens [14]. The same report also identified aminoglycosides as another empirical treatment regimen for infections with risk of MDR. In patients with complicated urinary tract infections or upper urinary tract infections who do not require hospitalization but for whom oral treatment options are limited due to resistance, aminoglycosides should be considered as the first-line treatment—provided that renal function is assessed and the dose is adjusted accordingly. In our study, the low resistance rates against gentamicin and amikacin across all regions indicated that aminoglycosides are a good empirical treatment option in Türkiye, as well. Parenteral aminoglycoside therapy (typically administered intramuscularly, such as amikacin or gentamicin) can be followed by oral step-down therapy with nitrofurantoin or fosfomycin once clinical improvement is achieved [13]. Although overall resistance rates to fosfomycin and nitrofurantoin were low, these are not recommended as the first-line treatment of complicated UTIs. However, the year-on-year increase in resistance trends, particularly for β-lactams and aminoglycosides, highlights the necessity of ongoing resistance surveillance to preserve the effectiveness of these antibiotics.

The high resistance rates against penicillins (30%), cephalosporins (> 30%) and ciprofloxacin (> 20%), represents a significant threat that limits empirical treatment options. Use of amoxicillin-clavulanate, ciprofloxacin and ceftazidime should be restricted to situations where no better alternatives are available, given their variable and often elevated resistance rates. Regional variability was also notable, with penicillin-class antibiotic resistance exceeding 60% in the Mediterranean and Eastern regions. The high resistance to third-generation cephalosporins, specifically ceftriaxone and ceftazidime, in these regions reflected elevated antibiotic consumption and inappropriate use and also raised serious concerns about the sustained efficacy of these agents. The regional difference on resistance is aligning with data from the Ministry of Health’s 2022 Health Statistics Yearbook, which highlights increased antibiotic use in Türkiye’s eastern and southeastern provinces [20, 21]. Particularly for the moderate-risk group with resistance rates between 20 and 40% (Fig. 5), treatment should be guided by urine culture and antibiotic susceptibility results. Encouraging this approach may help prevent further increases in resistance.

The Ministry of Health also recommends amoxicillin, amoxicillin-clavulanate, oral cephalosporins (such as cephalexin), fosfomycin, and nitrofurantoin for the treatment of cystitis and asymptomatic bacteriuria during pregnancy [13]. We observed 29.7% resistance to amoxicillin-clavulanate reaching as high as 53.3% in the Central East Anatolia region among all outpatient uropathogens isolated from women aged 18–45 years. The American College of Obstetricians and Gynecologists recommends avoiding amoxicillin-clavulanate due to increasing resistance patterns all over the world [22]. The latest study from Jordan reports amoxicillin-clavulanate resistance in pregnant patients as 42.4% [23], whereas amoxicillin-clavulanate resistance rates are reported 20.5% in Europe [24]. Given the high rates of resistance to beta-lactam antibiotics in Türkiye, nitrofurantoin or fosfomycin may be more reasonable as first-line options in pregnant patients as well, unless there is a suspicion for pyelonephritis. Gentamicin and ceftriaxone are also choices for treatment [22]. The low nitrofurantoin resistance rates for *E. coli* isolates observed in our study were consistent with international guidelines and supported the use of this antibiotic in pregnant women in Türkiye.

UTIs among elderly population particularly in nursing home residents became an emerging clinical challenge. The older patients with UTI usually present nonspecific signs and symptoms that might lead to misdiagnosis and misuse of antibiotics [25]. Unfortunately, the overuse of antibiotics in this population promotes antibiotic resistance and MDR acquisition in uropathogens, emphasizing the importance of considering patient age when selecting empirical therapy [26, 27]. In this study, the resistance rates against the majority of the antibiotics were notably high and MDR rate was higher in the elderly compared to the general population, exceeding 40% in some regions. These elevated rates are particularly concerning given the increased susceptibility of older adults to complicated urinary tract infections and other serious complications. The reliance on these agents for empirical therapy in Türkiye could be a serious risk for treatment failure or recurrent infections [28, 29]. Although resistance rates to nitrofurantoin and amikacin remained low, the low efficiency of nitrofurantoin in complicated cases, and potential nephrotoxicity of amikacin limits their use in this age group, thereby restricting therapeutic options. These findings underscore the urgent need for age-specific, regionally informed empirical treatment guidelines and robust antimicrobial stewardship strategies to mitigate further resistance development in this vulnerable population.

Ertapenem, a parenteral option suitable for treating complicated urinary tract infections in outpatients, has been unavailable in Türkiye for an extended period. This shortage has led to increased use of broader-spectrum carbapenems like meropenem and imipenem and unnecessary hospitalizations. This issue poses a significant barrier to effective antimicrobial stewardship in Türkiye. Although carbapenem resistance generally remained under the 20% threshold, higher rates particularly in *P. aeruginosa* and *K. pneumoniae* further complicates empiric therapy options for inpatients with potentially life-threatening infections.

Although *E. coli* remains the most common uropathogen in both outpatients and inpatients, consistent with global data [7, 30], *K. pneumoniae* infections are particularly concerning among inpatients due to their high resistance. *K. pneumoniae* is the leading cause of healthcare-associated infections in Türkiye [31–33]. These elevated resistance levels significantly increase the risk of treatment failure. Even broad-spectrum antibiotics face serious limitations: resistance rates to ceftriaxone (66.9%), and ciprofloxacin (60.7%) severely restrict empirical treatment options. Amikacin and gentamicin resistance were relatively low (25.7% and 33.3%, respectively) among the inpatient isolates. Alarmingly, resistance to ceftazidime-avibactam has exceeded 60%, threatening the efficacy of one of the few remaining last-line therapies [34]. Given the rising risk of antibiotic resistance among inpatients, the potential increase in ceftazidime-avibactam resistance is especially concerning (OR: 1.872). In line with current guidelines, including IDSA recommendations for cUTI [35], empirical therapy subsequently adjusted to targeted therapy based on culture and susceptibility results can be implemented in inpatients with UTIs.

In conclusion, urinary tract infections continue to pose a major public health challenge in Türkiye, particularly in light of rising antimicrobial resistance. This study highlights the critical need for up-to-date, regionally informed empirical treatment guidelines based on robust resistance surveillance data, coupled with a comprehensive stewardship program. While nitrofurantoin and fosfomycin remain effective options for uncomplicated infections in low-risk outpatients, their overuse must be carefully monitored. Aminoglycosides, with persistently low resistance rates, could be empirical options for infections with MDR risk, especially in inpatient settings. However, high resistance rates to commonly used antibiotics such as cephalosporins, and even last-line agents like ceftazidime-avibactam are deeply concerning. Pregnant women and elderly patients face additional risks due to reduced treatment options.

Overall, these findings emphasize the urgency for antimicrobial stewardship, reduced inappropriate antibiotic use, and the development of regional treatment protocols to prevent resistance increase and ensure effective management of UTIs in Türkiye. Future studies incorporating detailed clinical data and molecular characterization of resistance mechanisms are warranted to better inform treatment strategies, surveillance efforts and stewardship programs.

## Limitations

This study has the following limitations. First, its retrospective design may introduce inherent biases and should be considered when interpreting the results. Second, the analysis was based primarily on microbiological data, with limited clinical information available (restricted to age and gender). This may introduce some selection bias; however, the large sample size of > 100,000 positive urine cultures likely mitigates the impact of this limitation. Additionally, the analysis did not distinguish between uncomplicated and complicated UTIs, which may limit the clinical applicability of the findings to specific patient subgroups, although measures were taken to minimize the inclusion of recurrent infections. Finally, molecular mechanisms underlying antimicrobial resistance against, such as carbapenemase genes (e.g., *bla*_*NDM*_), were not investigated, particularly related ceftazidime–avibactam resistance, which may limit interpretation of resistance patterns at the genotypic level.

## Data Availability

No datasets were generated or analysed during the current study.
